# VfoldCPX Server: Predicting RNA-RNA Complex Structure and Stability

**DOI:** 10.1371/journal.pone.0163454

**Published:** 2016-09-22

**Authors:** Xiaojun Xu, Shi-Jie Chen

**Affiliations:** 1 Department of Physics, University of Missouri, Columbia, MO 65211, United States of America; 2 Department of Biochemistry, University of Missouri, Columbia, MO 65211, United States of America; 3 MU Informatics Institute, University of Missouri, Columbia, MO 65211, United States of America; Hong Kong University of Science and Technology, HONG KONG

## Abstract

RNA-RNA interactions are essential for genomic RNA dimerization, mRNA splicing, and many RNA-related gene expression and regulation processes. The prediction of the structure and folding stability of RNA-RNA complexes is a problem of significant biological importance and receives substantial interest in the biological community. The VfoldCPX server provides a new web interface to predict the two-dimensional (2D) structures of RNA-RNA complexes from the nucleotide sequences. The VfoldCPX server has several novel advantages including the ability to treat RNAs with tertiary contacts (crossing base pairs) such as loop-loop kissing interactions and the use of physical loop entropy parameters. Based on a partition function-based algorithm, the server enables prediction for structure with and without tertiary contacts. Furthermore, the server outputs a set of energetically stable structures, ranked by their stabilities. The results allow users to gain extensive physical insights into RNA-RNA interactions and their roles in RNA function. The web server is freely accessible at “http://rna.physics.missouri.edu/vfoldCPX”.

## Introduction

Many important biological processes such as mRNA splicing [1], microRNA-target recognition [2], and RNA-RNA dimerization [3] involve RNA-RNA interactions, including loop-loop interactions. Understanding such RNA functions requires an accurate tool to predict the structures and stabilities of RNA-RNA complexes. Methods seeking conserved RNA-RNA interactions through sequence comparisons [4–6] can be highly effective, but the approach relies on the existence of homologous sequences. Free energy-based physical models are not restricted by homologous sequences. However the approach is limited by the challenge of conformational sampling and the accuracy of energy parameters. Several physical models have been developed for RNA-RNA complexes with the different levels of constraints of the conformational spaces. For example, RNAhybrid [7] and UNAFold [8] ignore the intra-molecular base-pairing, and compute the minimum free energy (MFE) secondary structure with inter-molecular base pairs. These approaches tend to be more useful for shorter sequences, for which inter-strand base pairs can be more extensive than intra-strand base pairs. Other models, such as RNAcofold [9], PairFold [10], and IntaRNA [11] can treat both inter- and intra-strand base pairs for pseudoknot-free structures (i.e., base pairs do not cross). NUPACK [12] extends the single-stranded partition function algorithm to treat multiple interacting nucleic acid strands with a dynamic programming algorithm. HyperFold [13, 14], on the other hand, predicts multistrand nucleic acid complexes that can contain pseudoknots based on a novel search algorithm as well as a novel way to ascertain entropic contributions and kinetic accessibility. Other approaches such as RIP [15, 16], piRNA [17], bistaRNA [18], and RactIP [19], can treat more general RNA-RNA complex structures with tertiary (crossing) base pairs, such as pseudoknotted and hairpin-kissing motifs. However, the computational efficiency is notably lower than other models.

The folding of an RNA-RNA complex is govern by the same basic energetics as that of the intra-molecular folding of a one-strand RNA: base pairing and stacking energies and loop constraints [16]. Therefore, a straightforward approach [9, 10, 12] of folding two RNA molecules is to concatenate the two sequences and apply the same RNA-folding algorithm, with proper treatments for the connection region between the two strands. All these physical models rely on reliable energy/entropy parameters. For the secondary structures (2D structures containing no crossing base pairs), the nearest neighbor model with the assumption that stacking base pairs and loop entropies contribute additively to the free energy of RNA secondary structures [20–22] may be valid. However, for tertiary structures (whose 2D structures contain crossing base pairs), the folding free energy is nonadditive, i.e., the 2D structures can not be simply decomposed into helices and loops due to the correlation between them. For example, the stability of a loop is coupled to the helix due the loop-helix excluded volume and other interactions. As a result of the nonadditivity, the traditional recursive/backtracking algorithm fails, unless simplified energy models [23] that ignore the coupling/nonadditivity effects are used. The unphysical approximation about the thermodynamic parameters, in particular for tertiary motifs such as kissing loops, may contribute to the prediction inaccuracy.

Motivated by the demand for a thermodynamic model for RNA-RNA complexes, we have developed a new software and server (VfoldCPX) for the prediction of (2D) structures and the thermodynamic stabilities for RNA-RNA complexes. The thermodynamic parameters such as entropies and free energies in VfoldCPX are computed from a virtual bond-based RNA structure model (Vfold model). Through coarse-grained conformational sampling, the model gives the conformational entropy for the different types of kissing and pseudoknotted loop-loop motifs [24–28]. A unique advantage of the model is the ability to treat chain connectivity, excluded volume effect, and intra- and inter-molecular contacts. Using the loop free energy parameters from the Vfold model and the helix thermodynamic parameters from experiments, we predict the free energy landscape of RNA-RNA complexes, from which we determine the most stable and metastable structures from sequences. Extensive tests against the experimentally measured structure and thermodynamic data suggest that the Vfold-based loop parameters may be reliable [24–28].

## Methods

In the VfoldCPX algorithm, the input, two RNA sequences, are linked by a three-nucleotide phantom linker to transform the original two-RNA system into an effective one-RNA system, with proper treatment for the loops containing the phantom linker. For example, we should not assign entropy or enthalpy for a hairpin loop that contains the phantom linker because it is not a physical loop. Furthermore, the strand concentration-dependent free energy for the initiation of strand association is assumed to be independent of the RNA sequence. Therefore, all the RNA-RNA complex structures would have the same constant initiation energy for the binding of the two strands. In VfoldCPX, we do not include the constant initiation energy term in the total free-energy of RNA-RNA complexes. For a given structure, the VfoldCPX server computes the free energy for the helices based on two sets of thermodynamic parameters for base stacks: the Turner parameters [22] (04 version) and the MFOLD 2.3 version [29]. For the loop regions, the server employs the Vfold-calculated parameters. The current version of the server can treat loops with tertiary contacts such as pseudoknot loops and hairpin-hairpin kissing loop complexes [24–26]. The nonadditivity effect is accounted for because in the loop entropy calculation, loop conformations are generated in the context of the specific structural motif, i.e., the entropy and free energy parameters are motif-based. For example, pseudoknot loop conformations are sampled with the presence of the helix and the loop-loop kissing conformations are generated for the whole motif instead of individual loop. Here, we highlight only the main features of the algorithm. Further details can be found in the previously published papers [24–27].

### Structures without crossing base pairs (secondary structures)

To predict RNA-RNA complex structures within the secondary structure ensemble, we combine the recursive partition function calculation with the backtracking algorithm [30]. The partition function is computed through a recursive sum of the Boltzmann-weighted statistics over all the possible structures. The total partition function for the full chain is computed through a chain growth process. In each step, new base pairs are allowed to be added to the previous structures for the shorter chain.

**Fig 1:**
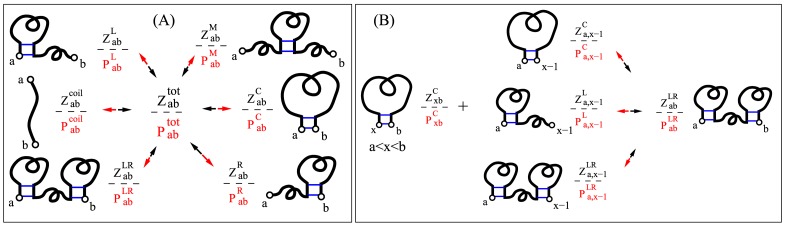
Schematic diagrams to show the recursive partition function calculation (in black) and the backtracking procedure (in red), for the total (A) and type *LR* (B) partition functions, respectively. For a given segment [a, b], we classify six types of conformations (*t* = *coil*, *C*, *L*, *R*, *LR*, *M*). The total partition function $Z_{\text{ab}}^{\text{tot}}=\sum_{\text{t}}Z_{\text{ab}}^{\text{t}}$. The backtracking begins with $P_{\text{1N}}^{\text{tot}}=1.0$ and proceeds differently for each type of conformational ensemble. As an example of the total conformational ensemble: the backtracking proceeds through $P_{\text{ab}}^{\text{t}}=(Z_{\text{ab}}^{\text{t}}/Z_{\text{ab}}^{\text{tot}})\cdot P_{\text{ab}}^{\text{tot}}$. Here, *N* is the RNA length and ${\text{Z}}_{\text{ab}}^{\text{coil}}=1.0$. The recursive relationship of other type of partition functions is shown in S1 Data

To account for the conformational compatibility in each conformational growth step, we classify the conformational ensemble into six types. Specifically, for each segment from nucleotides *a* to *b*, we define conformational types (*t* = coil, C, L, R, LR and M) according to the base pairing situations at the terminal nucleotides *a* and *b* (see Fig 1). The *coil* state is the one without any base pairs and its partition function is ${\text{Z}}_{\text{ab}}^{\text{coil}}=1.0$. Type *C* is the ensemble of conformations with (*a*, *b*) base paired. Type *L* (*R*) is the ensemble of conformations with nucleotide *a* (*b*) forming base paired with other nucleotide but *b* (*a*), respectively. Type *LR* is the ensemble of conformations with both nucleotide *a* and *b* forming base paired with other nucleotides but not with each other. And type *M* is the ensemble of conformations containing at least two base pairs while both *a* and *b* are unpaired. The six conformational types follow different recursive rules [31–33]; See Fig 1 and the Supplementary Information (S1 Data) for details. The total partition function is given by $Z_{\text{ab}}^{\text{tot}}=\sum_{\text{t}}Z_{\text{ab}}^{\text{t}}$. By tracing back how the total partition function $Z_{1\text{N}}^{\text{tot}}$ (for the full sequence from nucleotide 1 to nucleotide N) is calculated, we can recursively calculate the base pairing probabilities and the probable structures.

Our algorithm distinguishes itself from other models by classifying the different conformational types and hence accounting for the conformational connectivity more accurately. For example, when two helices are linked by a loop of < 2 unpaired nucleotides, we can add a coaxial stacking energy term to account for the real structural effect on the free energy calculations. The approach can account for the conformational compatibility due to constraints such as excluded volume and hydrogen bonding. As a result, the algorithm may provide an improved estimation for the overall conformational entropy and free energy.

### Structures with crossing base pairs (tertiary structures)

**Fig 2:**
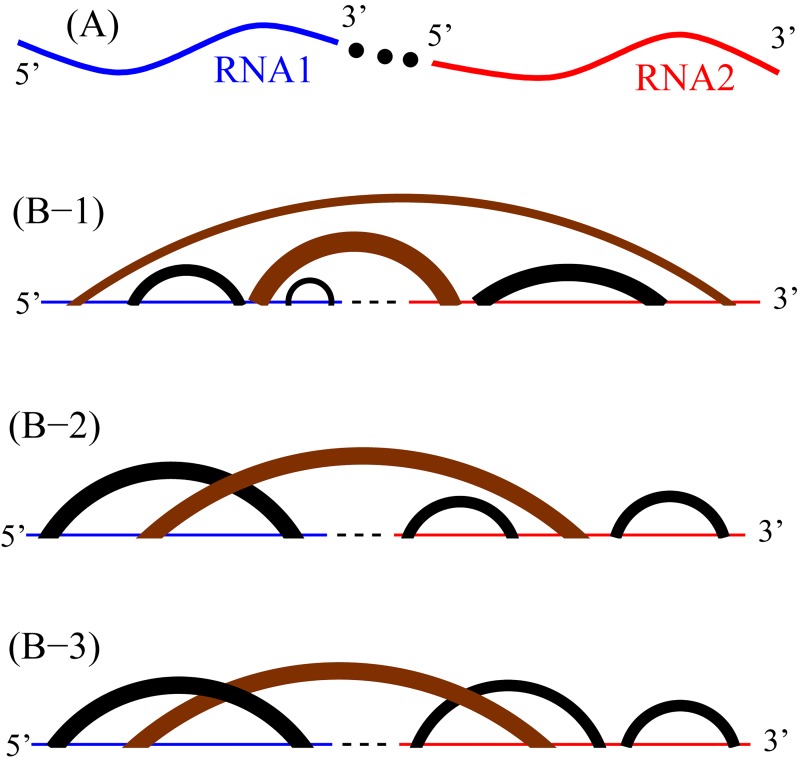
RNA-RNA complex system and three structural ensembles. (A) The input of two RNA sequences are linked by a three-nucleotide phantom linker to transform the original two-RNA system into an effective one-RNA system. (B) Three different structural types: (B-1) secondary, (B-2) H-type pseudoknotted, and (B-3) hairpin-hairpin kissed structures. The curved links in the diagrams denote base pairs (helix stems).

**Fig 3:**
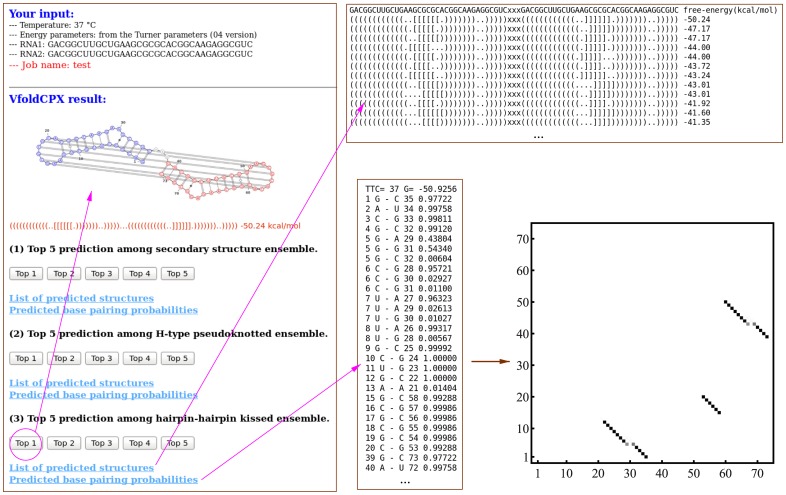
A snapshot of the output of the VfoldCPX server. Based on the total length of the input effective one-RNA system, the server provides up to three sets of predicted structures, corresponding to the three structural ensembles shown in Fig 2(B). In this example, the predicted most probable 2D structure (plotted using VARNA [35]) has the free energy of -50.24 kcal/mol. The predicted base pairing distributions shown by the density plot and the alternative stable structures provide important information about the structures and stabilities.

Because the current Vfold-predicted loop entropy parameters are available for only a limited number of loop types [24–28] and the inclusion of the crossing base pairs can lead to a significantly larger number of conformations, the current version of the VfoldCPX treats only medium-sized RNA-RNA complexes for structures with the crossing base pairs shown in (Fig 2B-2 and 2B-3). In S1 Data, we show the RNA sequence length-dependence of the computational time.

To enhance the computational efficiency, we use a two-step screening process to sample and rank RNA-RNA complex structures with crossing base pairs. In the first step, we sample the intermolecular crossing base pairs. We assume the crossing base pairs form a single helix stretch. We use this intermolecular helix to denote the binding site/mode “B” of the RNA-RNA complex (see the brown helices in (Fig 2B-2 and 2B-3)). We allow a (1×1) internal loop or a 1-nt bulge loop to be formed in this intermolecular helix stem. For each crossing base pair mode “B”, we use the recursive/backtrack algorithm to sample the rest non-crossing intra- and inter-molecular base pairs. The non-crossing base pairs form secondary structures thus the computation can be quite efficient with the secondary structure algorithm above. The sum of the statistical weight of all the sampled structures gives the partition function of mode B Z^B^. The mode B of the largest Z^B^ is the most probable mode.

In the second step, we run calculation only for the most probable mode B (or the top few most probable modes). Specifically, we use the above mentioned backtracking algorithm to predict the base pairing probability for the (non-crossing) inter- and intra-molecular base pairs ${\text{p}}_{\text{ij}}^{\text{B}}$ for all the allowed (*i*, *j*) pairs: ${\text{p}}_{\text{ij}}^{\text{B}}=Z_{\text{ij}}^{\text{B}}/Z^{\text{B}}$. Here, ${\text{Z}}_{\text{ij}}^{\text{B}}$ is the partition function for all the structures that contain base pair (*i*, *j*) and crossing inter-molecular helix B (see S1 Data). For this step, because we need to compute the base pairing probability of all the possible base pairs, the computation can be time-consuming.

## Results

### VfoldCPX input

The input of VfoldCPX is two sequences for the two RNA strands, respectively. Besides the temperature, users have the option to use the base stacking energy parameters either from Turner’s parameters or from the MFOLD parameter set. Based on the total length of the effective one-RNA system L_*tot*_ (the sum of the lengths of the two strands), the VfoldCPX server generates up to three sets of predicted RNA-RNA complex structures, as well as the base pairing probabilities:

1. L_*tot* ≤_ 300 nt for the secondary structure ensemble.
2. L_*tot* ≤_ 150 nt for the secondary, and H-type pseudoknotted structure ensemble.
3. L_*tot* ≤_ 120 nt for the secondary, H-type pseudoknotted, and hairpin loop-loop kissing structure ensemble.

### VfoldCPX output

Once a calculation is submitted, a notification page containing the job information, such as the job name, email address (optional), and the job status, is displayed. If an email address is provided by the user, when the calculation is finished, the VfoldCPX web server sends out an email notification with the predicted results attached. A user can either bookmark the job-specific notification page for checking the job status or keep the page in the browse window as the notification page is automatically updated as the job is finished.

Fig 3 shows an example of VfoldCPX prediction for the SL1-SL1 complex in HIV [34]. From the three sets of the predicted structures, we find two distinct binding interactions: the linear dimer and the hairpin loop kissing dimer. Based on the predicted free energies, the kissing dimer (produced by VARNA [35] in Fig 3) is the most probable structure. It is important to note that the predicted structures may not always correspond to the native ones. One reason is due to the uncertainty of the energy parameters derived from the experiments and the theory, such as the Vfold model for the RNA loop parameters [31]. Furthermore, an RNA complex may fold into alternative structures with similar stabilities in order to perform different roles in function. Therefore, VfoldCPX outputs a set of energetically stable structures (instead of a single structure) ranked by their stabilities and the base pair distributions, as shown in Fig 3 as an example. The results may help users to gain physical insights into RNA-RNA interactions and their biological functions.

## Conclusion

We have developed the VfoldCPX software and web server to predict RNA-RNA complex structures and folding thermodynamics. The web server provides a platform for the application of our continuously developed Vfold-based algorithms for the folding of RNA complexes. Currently, VfoldCPX can only treat RNA-RNA complex structures with at most one inter-molecular crossing base pairing helix. In the further development, VfoldCPX will be upgraded to treat RNA-RNA complexes with multiple binding sites, such as the fhlA/OxyS complex [36], which involves two simultaneous binding sites.

## Supporting Information

S1 Data The recursive relationship of partition functions, and the RNA sequence length-dependence of the computational time. (PDF)
