# Supplementary material for: VfoldCPX Server: Predicting RNA-RNA Complex Structure and Stability
Source: PLoS One. 2016 Sep 22;11(9):e0163454. doi: 10.1371/journal.pone.0163454 (PMC5033388; doi:10.1371/journal.pone.0163454)
Supplement: S1 Data — (PDF) [file pone.0163454.s001.pdf]

Supplementary Information:

VfoldCPX server: Predicting RNA-RNA complex structure and stability

Xiaojun Xu, and Shi-Jie Chen
Department of Physics, Department of Biochemistry, and Informatics Institute
University of Missouri, Columbia, MO 65211

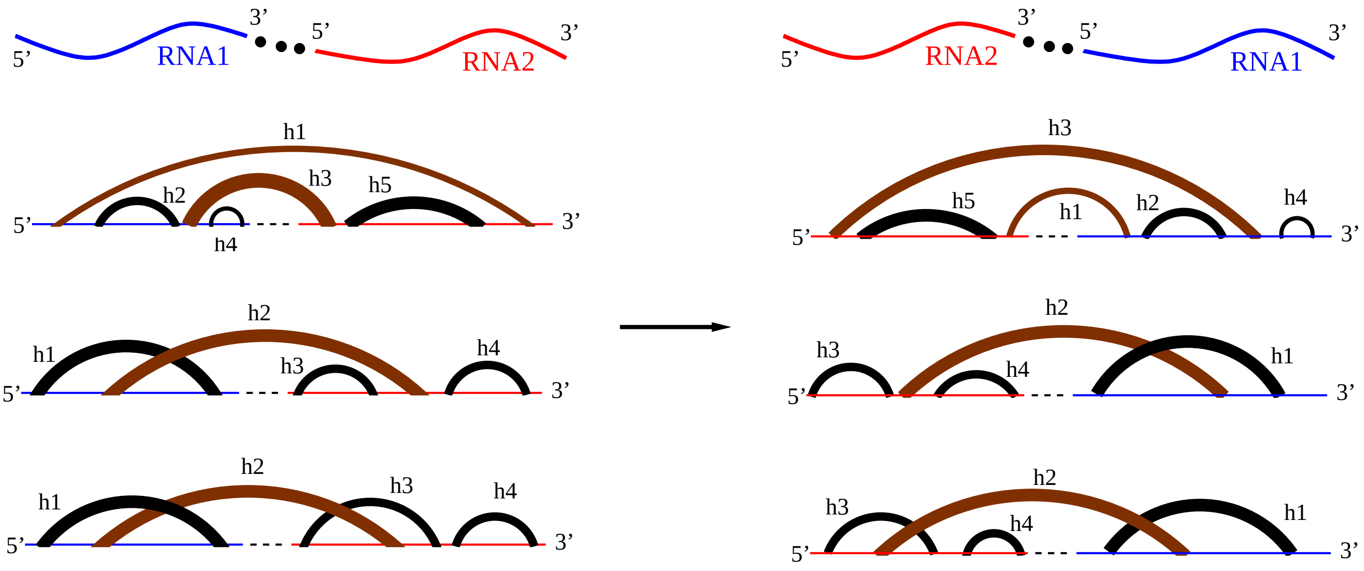

Figure S1: Two different orders for connecting two RNA sequences into one RNA system by a three-nucleotide phantom linker. The change of the order does not change the structure types for the three structure ensembles in the VfoldCPX calculations.

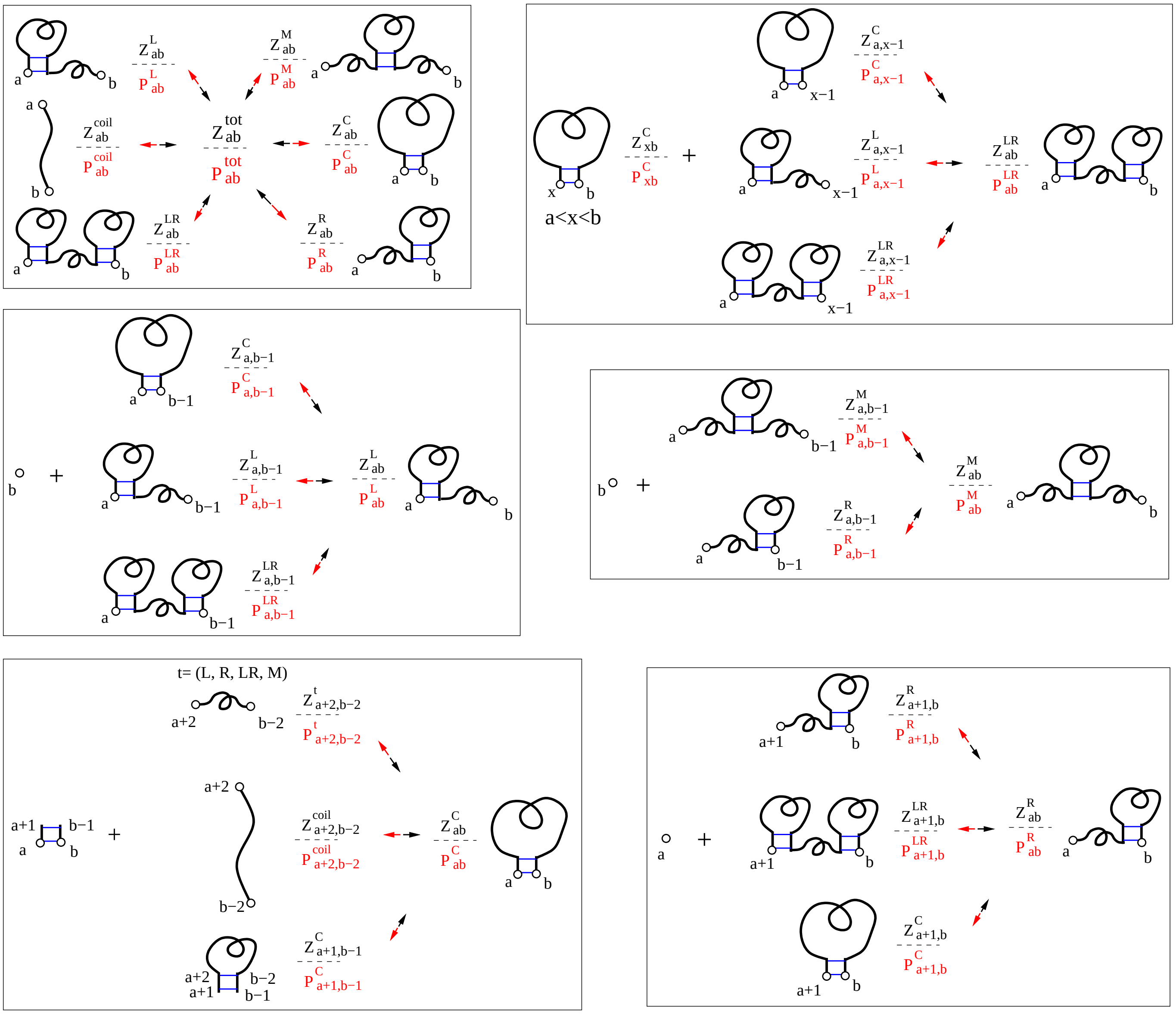

Figure S2: For a given segment [a,b], we classify six types of conformations. The *coil* state is the one without any base pairs. The type C is the ensemble of conformations with (a,b) base paired. The type L/R is the ensemble of conformations with nucleotide a/b base paired with other nucleotides (except b/a). The type LR is the ensemble of conformations with both nucleotide a and b forming base paired with other nucleotides but not with each other. And the type M is the ensemble of conformations containing at least two base pairs, but both a and b are unpaired. The total partition function is the sum over all the six types of conformations ($\mathrm{t} = coil, C, L, R, LR, M$): $Z^{\mathrm{tot}}_{\mathrm{ab}} = \sum_{\mathrm{t}} Z^{\mathrm{t}}_{\mathrm{ab}}$. The backtracking process starts from $P^{\mathrm{tot}}_{1\mathrm{N}} = 1.0$ and proceeds differently for each type of conformational ensemble. As an example of the total conformational ensemble, the backtracking proceeds through $P^{\mathrm{t}}_{\mathrm{ab}} = (Z^{\mathrm{t}}_{\mathrm{ab}}/Z^{\mathrm{tot}}_{\mathrm{ab}})\cdot P^{\mathrm{tot}}_{\mathrm{ab}}$. Here, N is the RNA length and $Z^{\mathrm{coil}}_{\mathrm{ab}} = 1.0$.

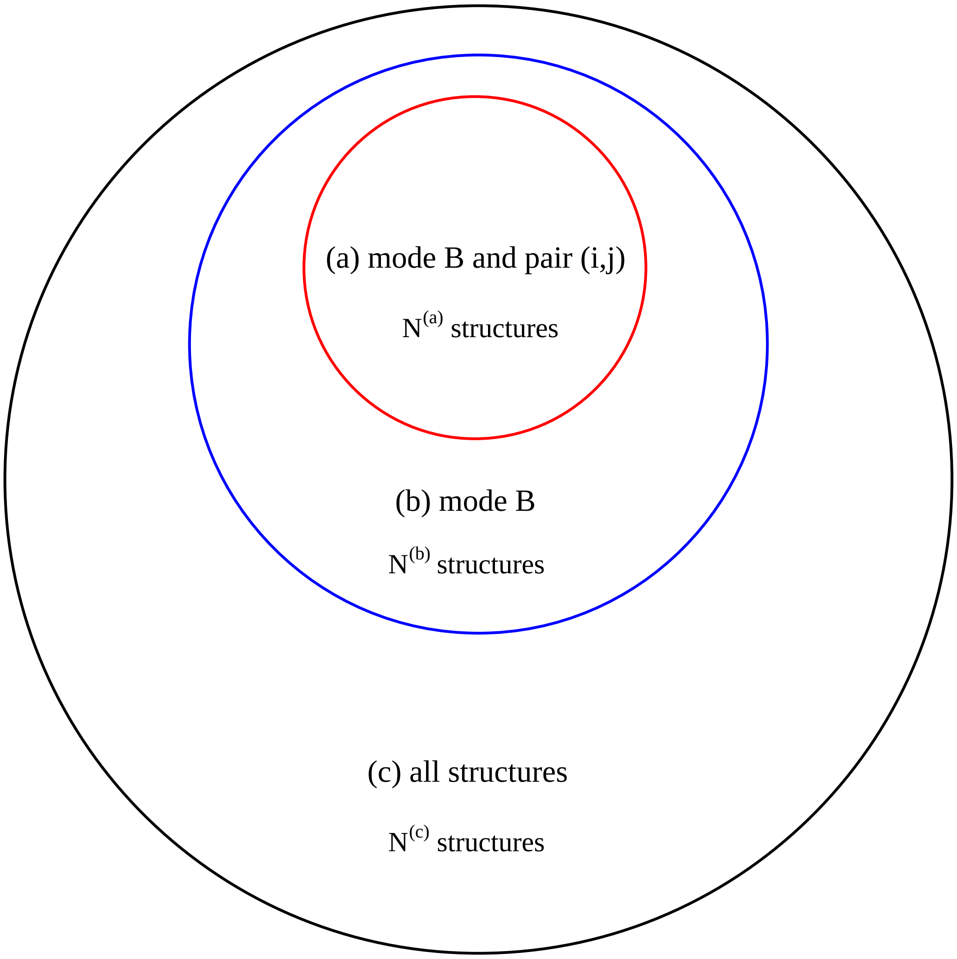

Figure S3: Structural ensembles and (conditional) partition functions. (a) The ensemble of totally $N^{(a)}$ structures with the binding mode ”B” and the (i, j) base pair. The (conditional) partition function $Z_{ij}^{B} = \sum_{s=1}^{N^{(a)}} e^{-G(s)/k_B T}$. Here, G(s) is the free energy of structure s. k_B is the Boltzmann constant and T is the temperature. (b) The ensemble of $N^{(a)}$ structures of the binding mode ”B”, including $N^{(a)}$ and $(N^{(b)}$-$N^{(a)})$ structures with and without the (i, j) base pair, respectively. $Z^{B} = \sum_{s=1}^{N^{(b)}} e^{-G(s)/k_B T}$. (c) The ensemble of all the $N^{(c)}$ structures with all the possible binding modes. $Z^{tot} = \sum_{s=1}^{N^{(c)}} e^{-G(s)/k_B T}$ gives the total partition function. $P^{B} = Z^{B}/Z^{tot}$ denotes the population of all the structures with the binging mode “B”. $P_{ij}^{B} = Z_{ij}^{B}/Z^{tot}$ gives the population of all the structures with the binding mode ”B” and the (i, j) base pair.

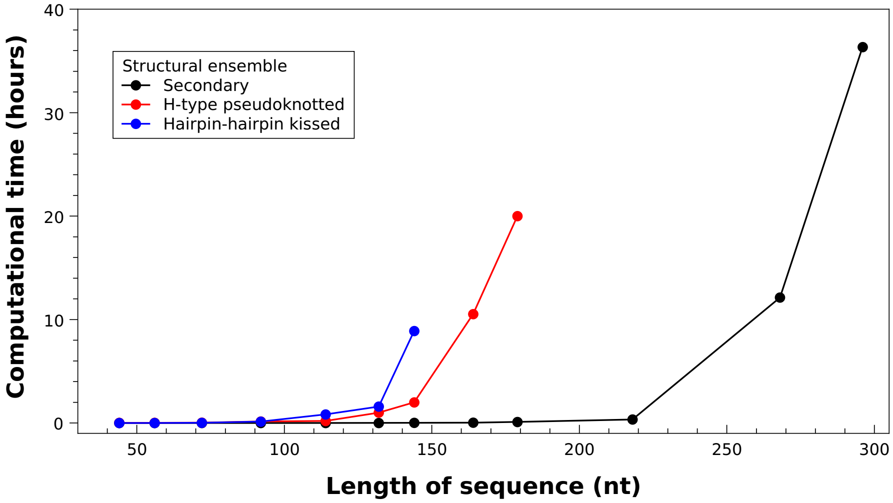

Figure S4: Computational time of VfoldCPX for the ensemble of the secondary structures only (in black), ensemble including H-type pseudoknotted structures (in red), and the ensemble including hairpin-hairpin kissing structures (in blue). The computer resource used in the test is HP pavilion HPE with the Intel (R) Core (TM) i7-2600 (3.40 GHz) processor.
